# Personalized Digital Care Program Allocation for Older Adults: Reinforcement Learning-Based Simulation Study

**DOI:** 10.2196/86710

**Published:** 2026-04-13

**Authors:** Hongsoo Kim, Kyungbok Lee, Jae Yoon Yi, Myunghee Cho Paik

**Affiliations:** 1 Department of Public Health Sciences Graduate School of Public Health Seoul National University Seoul Republic of Korea; 2 Artificial Intelligence Institute Seoul National University Seoul Republic of Korea; 3 Institute of Health and Environment Seoul National University Seoul Republic of Korea; 4 Department of Biostatistics Gillings School of Global Public Health University of North Carolina at Chapel Hill Chapel Hill, NC United States; 5 Shepherd 23 Inc. Seoul Republic of Korea

**Keywords:** precision digital care, adaptive program assignment, contextual bandits, AI-enabled care, artificial intelligence, aging population, simulation study

## Abstract

**Background:**

As the demand for innovative older adult care grows alongside a shortage of care workers, personalization is key to optimizing services and enhancing long-term sustainability. This study proposes an adaptive reinforcement learning (RL)-based framework to promote precision digital care by dynamically assigning care programs based on individuals’ unique characteristics and evolving needs. Its effectiveness was evaluated through simulation-based experiments comparing multiple allocation methods within an artificial intelligence (AI)-powered care call service for older adults.

**Objective:**

This study aimed to develop and evaluate an RL-based model for personalizing digital care program allocation to optimize care engagement and health outcomes among low-income older adults living alone.

**Methods:**

We developed the framework by using contextual bandits, specifically Thompson Sampling, to maximize user outcomes. Four program allocation strategies were tested using a synthetic dataset of user features and program attributes. Simulations were conducted over multiple iterations to evaluate how the model adapts over time and optimizes program assignments compared with static methods.

**Results:**

Four program allocation methods were compared across 100 simulation runs (n=3000 assignments per run) using 2 datasets (AI Call: n=1196; Community Health Survey [CHS]: n=72,812): (1) systematic allocation (baseline), (2) single best program based on population average, (3) idealized personalized delivery (theoretical upper bound), and (4) precision digital care using Thompson Sampling. Precision digital care outperformed baseline and population-average approaches, achieving outcomes comparable to the theoretical upper bound. Compared to systematic allocation, call success rates increased by 84.2% (AI Call) and 54.4% (CHS), Patient Health Questionnaire-2 depression scores decreased by 32.1% (AI Call) and 41.4% (CHS), and self-reported health scores improved by 19% (AI Call) and 22% (CHS). It also showed improved learning efficiency, refining assignments dynamically as it learned from user responses.

**Conclusions:**

Our findings emphasize the importance of personalization in digital care. We plan to refine and validate the model through a publicly funded AI care program for community-dwelling, low-income older adults living alone in the Republic of Korea. RL offers a scalable and effective approach to advance precision digital care delivery and support future innovations in aging services.

## Introduction

The rapid aging of populations and increasing life expectancy demand integrated care and health services to support individuals throughout extended later life. Preventive care, aimed at promoting independent living and delaying institutionalization, has emerged as a key strategy in aging policies across developed countries [1]. Citizens increasingly expect robust social protection from their governments. Meanwhile, health and care workers are grappling with severe burnout and workforce shortages [2,3]. Despite numerous social policy reforms, traditional approaches to addressing the growing “care crisis” are proving inadequate. The failures of both policy and market-based strategies to protect vulnerable older adults during the COVID-19 pandemic underscore the urgent need for transformative changes to achieve global healthy aging [4].

Technological innovation is widely recognized as a powerful solution to the social risks posed by demographic shifts [5,6]. The COVID-19 pandemic catalyzed expansions in R&D and market investments, accelerating the transition from lab-based innovation to real-world applications, such as the widespread adoption of telemedicine [7]. In the postpandemic era, breakthroughs like GPT and large language models have ushered in a new age of digital health and care, opening unprecedented opportunities.

Within this digital health landscape, precision medicine has garnered significant attention [8]. The integration of big data from electronic medical records has propelled advancements in precision medicine, driving the development of personalized medical diagnostics and treatments [9,10]. These innovations hold immense promise, from curing previously untreatable diseases to rapidly discovering new drugs and materials. Consequently, many countries are actively investing in R&D and fostering global cooperation through public-private partnerships.

In contrast, the personalization of care services for older adults has received comparatively limited attention, despite significant progress in digital care for older adults. While precision medicine has primarily focused on genomics, biomarkers, and individualized treatment plans in clinical settings, precision care addresses broader contextual factors, including behavioral data, social determinants, and real-world service use. Precision medicine leverages structured biological data to optimize therapeutic decisions, often within highly controlled environments such as hospitals or labs. In contrast, precision care operates in complex, community-based settings and must contend with heterogeneous data types, evolving patient preferences, and ethical imperatives related to autonomy and accessibility.

We think precision care represents a paradigm shift that expands the individualization principle beyond disease treatment to service delivery and engagement support, especially in aging and chronic care domains. Therefore, the transition from precision medicine to precision care in this study reflects a shift in context, data sources, ethical priorities, and delivery models, rather than a simple semantic change. Several challenges impede this shift, including insufficient large-scale, high-quality care data, lack of data standardization, decentralized data governance, and fragmented health and care systems [11,12]. However, promising initiatives, such as the EU-funded iCare4Old project, demonstrate how these barriers can be overcome [13]. Such projects exemplify the potential of cross-national, standardized, comprehensive geriatric assessment databases linked to mortality and health care use data to support decision-making for older adults with complex chronic conditions.

Digital care is also a high policy priority in Korea, an East Asian country that is expected to face the most severe shortage of care workers among Organization for Economic Co-operation and Development countries by 2040 [2]. Various government-funded digital health and care programs have been developed and implemented to help delay cognitive decline and alleviate loneliness, with their delivery managed through diverse local government units. For example, the central agency for social services in Korea provides artificial intelligence (AI)-based care calls for older adults living alone for checking in and providing information [14]. Although still in the early stages of implementation both technologically and in terms of service design, many social welfare institutions have adopted the AI care call service to alleviate the overtime workload of direct care workers and to prevent unattended deaths among the older adults, an emerging social problem in Korea. The local government, in collaboration with interest groups from universities or industries, implements the Care Call program developed by these groups. While many programs are created through partnerships with local governments, users do not have a choice of programs; instead, the program they receive is determined by their location. As these programs expand to larger populations, they must be evaluated and delivered efficiently.

While AI-driven digital care is expanding, current approaches rely on rule-based or non-adaptive personalization, which may fail to optimize interventions dynamically at the individual level. In health care and digital interventions, the term personalization often refers to the adaptation of care strategies based on characteristics shared within predefined subgroups, such as age, gender, or geographic location. Individuals within these homogeneous groups receive the same intervention, under the assumption that group-level traits sufficiently capture variation in needs or responses. However, this approach can overlook meaningful individual differences. In contrast, hyper-personalization refers to a finer-grained strategy in which care is tailored at the individual level based on contextual signals such as prior responses, behavioral patterns, or dynamically changing health indicators. Rather than assigning treatments to groups, hyper-personalized care selects the best intervention for each instance, enabling adaptive learning and improved outcomes. Our work embraces this hyper-personalization framework by using contextual bandits to deliver care content that evolves with each person’s unique context and feedback history.

Beyond technical optimization, hyper-personalization addresses a fundamental challenge in digital care for older persons: one-size-fits-all approaches often lead to disengagement, frustration, and abandonment among older adults [15]. Older people vary widely in cognitive capacity, health literacy, communication preferences, and daily routines [16]. Receiving care content misaligned with these individual contexts can feel impersonal, patronizing, or irrelevant, undermining both engagement and dignity. For example, an older adult with mild cognitive impairment may benefit most from brief, frequent check-in calls, while someone experiencing loneliness may prefer longer, conversational interactions. By tailoring content to individual context and response patterns, precision digital care respects individual autonomy and maintains dignity through person-centered service delivery.

We model the care call assignment as a repeated decision-making problem, where the system observes individual-level context, selects a care message (action), receives feedback, and uses it to improve future decisions. This setting goes beyond supervised learning, which does not handle the need for sequential adaptation or balance between exploration and exploitation. While Q-learning and other reinforcement learning (RL) methods can technically be applied, they are designed for environments where actions influence future states—an unnecessary assumption in our context, where each care interaction is a standalone event with immediate feedback and does not affect the next person’s reward. Contextual bandits with Thompson Sampling offer a natural, data-efficient, and interpretable approach for personalizing care at scale, without introducing unnecessary complexity.

This study proposes precision digital care as an RL-based framework using contextual bandits, specifically designed to personalize care services dynamically. This approach assigns care programs to individuals based on their unique characteristics and evolving care outcomes, continuously optimizing decisions over time. At its core, the framework leverages contextual bandits, a type of RL algorithm, specifically Thompson Sampling [17], to balance exploration and exploitation in digital health interventions. This computational approach enables the model to learn from user interactions, adapt in real time, and continuously refine program assignment decisions to maximize individual outcomes. Unlike traditional rule-based or static personalization methods, the proposed precision digital care approach dynamically optimizes care allocation, ensuring interventions remain responsive and effective.

By integrating Thompson Sampling, the proposed approach provides a scalable, data-driven solution for enhancing personalization, efficiency, and equity in digital health services, particularly in aging care. To evaluate its effectiveness, this study uses simulation-based experiments comparing multiple program allocation methods and analyzing their impact on key health and service-related outcomes. This study proposes precision digital care as an RL-based model designed to enhance quality of life in later years by delivering personalized, humane, and adaptive care. Rather than simply maximizing technical metrics, our approach uses RL as a tool to improve care engagement and health outcomes, supporting autonomy, reducing emotional distress, and ultimately enabling older adults to age in place with dignity. Importantly, by minimizing cumulative regret during the learning process, the approach reduces exposure to suboptimal care while enabling adaptive, sequential learning even in data-sparse or dynamically changing environments**.** By evaluating this approach through simulation, we aim to provide evidence for improving both efficiency and meaningful outcomes for vulnerable older adults before real-world implementation.

## Methods

### Aim of Simulations

In Korea, researchers are actively developing a range of government-funded digital care programs aimed at improving the well-being of older adults. These programs are administered by local authorities and are currently tailored to specific groups of older adults in distinct regions. As these initiatives progress, the accumulation of knowledge and data is expected to pave the way for large-scale implementation in the near future. This study focused on the critical role of personalization in enhancing the effectiveness of digital care. While personalization has not yet been fully integrated, demographic data on recipients of digital care programs is available. Leveraging this information, we conducted simulation studies that modeled the baseline characteristics of user populations, simulating the potential impact of personalized interventions.

### Simulation Methods

To evaluate the potential of personalized digital care, we conducted a series of simulations mimicking the delivery of care programs to older adult residents in a community setting.

#### Four Allocation Methods

Our simulations were designed to evaluate the method that optimizes the outcome of interest. These 4 allocation methods were selected to represent commonly used approaches in digital health intervention deployment, ranging from traditional rule-based methods (systematic) to theoretically optimal but not feasible in practice (idealized personalized). By comparing these methods, we assessed the added value of the RL-based precision digital care approach in real-world care allocation settings.

#### Method 1: Systematic Delivery (Baseline)

This method reflects a common real-world practice, including the Korean local government model, where programs are assigned to users in a fixed order, without considering individual needs or program characteristics. This approach offers the simplest implementation, requiring no information about individual users or program effectiveness. However, it represents a naive baseline that can lead to suboptimal outcomes by ignoring potential benefits from personalized program allocation.

#### Method 2: Single Best Program (Theoretical Benchmark)

This method simulates a hypothetical situation where we know the single best program for the entire population based on average outcomes. This method mimics a scenario where the result of a large-scale randomized controlled trial, where individual characteristics are not used in program assignment, is known. This method provides a useful benchmark for comparison. It demonstrates the upper limit of effectiveness achievable when using a single program for everyone and highlights potential limitations of a one-size-fits-all approach. If there were no individual variability, the one-size-fits-all approach would be optimal.

#### Method 3: Idealized Personalized Delivery (Theoretical Upper Bound)

This method assumes perfect knowledge of each individual's optimal program, representing an ideal but unattainable scenario in real-world settings. This method provides a theoretical upper bound on personalization benefits, demonstrating the best possible outcome achievable if we could perfectly predict the most effective program for each individual. Comparing this method to others highlights the potential gains from personalization.

#### Method 4: Precision Digital Care with Thompson Sampling (Personalized Approach)

This method addresses the challenge of sequentially assigning programs to individuals without prior knowledge of which program will be most effective for each person. This problem can be framed as a “sequential decision making under uncertainty” problem, where the goal is to maximize cumulative rewards over time. To tackle this, we used a contextual bandit framework, specifically using the linear Thompson Sampling algorithm [17].

In this setup, each care program is treated as an “arm” in the contextual bandit framework, where the reward function represents the expected benefit for a given user. The model dynamically updates its understanding of which arm (care program) is most effective based on observed user responses, allowing for personalized interventions over time. The relationship between the outcome (reward) and the context (individual characteristics and program features) is unknown and must be estimated as users experience the program. The challenge lies in balancing exploration (trying different programs to estimate or learn their effectiveness) and exploitation (assigning the program with the highest estimated reward based on current knowledge).

Among various contextual bandit algorithms, Thompson Sampling offers an effective approach for this setting. While upper confidence bound algorithms select arms by calculating an upper confidence bound on their estimated rewards, Thompson Sampling is motivated by a Bayesian approach. Specifically, it draws from the posterior distribution of each arm's reward, and the arm with the highest sampled reward is then selected.

This randomized approach effectively balances exploration and exploitation. In early stages, when uncertainty is high, the algorithm explores a wider range of programs due to the variability in sampled rewards. As more data is collected, the posterior distributions become more concentrated around the true reward values, and the algorithm increasingly exploits the program with the highest estimated reward for each individual. This approach enables precision digital care to adapt to individual differences and learn the optimal program assignment over time.

#### Data-Generating Mechanisms

To conduct simulation studies, we specified the distribution of the baseline user characteristics and the relationship between these characteristics and the outcomes. Our study involved a total of 6 distinct simulation scenarios, evaluating 3 different outcome measures (detailed in “Setup for Outcome Performance Measures”) under 2 different assumptions about the user population and data availability, each informed by a specific Korean data source. The process for establishing these simulation environments was described below.

The first set of simulations aimed to closely mimic the characteristics and responses observed in a specific digital care intervention context. This was informed by data collected as part of a primary study involving approximately 1000 older adults actively engaging with an AI-based care call service (the “AI Call dataset”). This dataset provided detailed individual-level information on demographics, health status, intervention parameters (call success), and key outcomes (loneliness and self-rated health).

The second set of simulations aimed to represent a broader, more general population of older adults, reflecting demographic distributions found in large-scale surveys but potentially lacking detailed intervention-specific outcome data. This was informed by a representative sample from the national Community Health Survey (CHS) conducted in 2020 [18]. This survey provided robust population-level distributions for key demographic, health, and psychosocial variables. Table 1 describes the baseline distribution of key independent and dependent variables across the 2 datasets.

By comparing results across these 2 sets of simulations (AI Call dataset-based and CHS-based) for each outcome measure, we aimed to assess the robustness of our findings under different data conditions. The following sections detail how user profiles and the outcome generation process were specifically constructed for each simulation type.

**Table 1:** Baseline distribution of the key dependent and independent variables.

Variables				AI^a^ Call dataset (n=1196)		CHS^b^ dataset (n=72,812)
**Independent variables**						
	**Age, n (%)**					
		Young-old (65-74 years)	242 (20.2)		38,590 (53)	
		Old-old (≥75 years)	954 (79.8)		34,222 (470)	
	**Sex, n (%)**					
		Male	273 (22.8)		30,326 (41.7)	
		Female	923 (77.2)		42,486 (58.4)	
	**Region, n (%)**					
		Urban	342 (28.6)		29,846 (41)	
		Rural	854 (71.4)		42,966 (59)	
	**Loneliness, n (%)**					
		Yes	136 (11.4)		4660 (6.4)	
		No	1060 (88.6)		68,152 (93.6)	
**Dependent variables**						
	**Self-rated health, n (%)**					
		Very good	20 (1.7)		3073 (4.2)	
		Good	165 (13.8)		20,577 (28.3)	
		So-so	630 (52.7)		28,746 (39.5)	
		Bad	341 (28.5)		15,975 (21.9)	
		Very bad	39 (3.3)		4441 (6.1)	
	**Depression, n (%)**					
		Nondepressed	923 (77.2)		70,264 (96.5)	
		Depressed	273 (22.8)		2548 (3.5)	
Call success rate, mean (SD)				0.86 (0.35)		—^c^

^a^AI: artificial intelligence.

^b^CHS: community health survey.

^c^Not applicable.

### Setup for Baseline Distribution

To investigate the potential of personalized program allocation under different scenarios, we constructed synthetic user profiles representative of the 2 populations described above. User profiles were defined by a combination of 7 binary demographic and health-related features: age (older or younger), sex (male or female), region (rural or urban), economic status (unstable or stable), presence of multiple chronic conditions (present or absent), functional difficulty (present or absent), social isolation (high or low), cognition (intact or not intact), and living arrangement (alone or with others). The generation process differed depending on the simulation set:

For simulations based on the AI Call dataset: Synthetic user profiles were generated by sampling directly from the characteristics of about 1000 users in the raw AI Call dataset. This ensured the simulated users closely mirrored the specific population engaging with that service. For simulations based on the CHS dataset: since only aggregated distributional information (eg, percentage breakdowns for age, sex, and region) was available from the CHS summary data, synthetic user profiles were generated differently. Each user characteristic was randomly generated independently according to the proportions reported in the CHS data. This method produced a synthetic population whose overall marginal distributions matched the CHS population structure, assuming independence between characteristics based on the available information. Similarly, we created a pool of programs with varying attributes (content type, frequency, duration, and voice type). Parameters defining individuals (based on the methods above) and the range of programs were set to represent typical variations observed in Korean older adult care contexts.

### Setup for the Relationship Between Reward and Context

A critical component defining each simulation scenario is the relationship between a user's characteristics, the assigned program attributes, and the resulting outcome or reward. This relationship models the differential program effectiveness, which personalization aims to exploit. The construction of this relationship also differed between the two simulation sets: (1) For simulations based on the AI Call dataset, the parameters (coefficients) governing the relationship between user and program features and outcomes were informed by observed patterns in the actual AI Call data. Specifically, the direction (sign) of main effect coefficients was set to match observed trends (eg, whether rural users in the data had higher or lower call success). For simplicity, the magnitude of these data-informed coefficients was held constant; and (2) for simulations based on the CHS, since the CHS data lacked information on how user characteristics relate to outcomes from specific digital care programs, the coefficients defining the relationship between user and program features and outcomes were assigned randomly. This simulated outcome variability without imposing specific assumptions based on the limited data.

In both simulation sets, to explicitly model the potential for personalization, several user-program interaction effects were included. These were based on domain knowledge and plausible scenarios. Importantly, these interaction effect scenarios were applied consistently across all 3 outcome measures. Specific examples of modeled interactions include scenarios where:

1. Women derived higher rewards from longer calls.
2. Users with functional limitations benefited more from longer calls.
3. Rural users preferred more frequent calls.
4. Those with older age preferred check-in calls.
5. Those with low cognitive function preferred check-in calls.
6. Lonely individuals preferred intervention calls less.
7. Those with chronic disease preferred health information calls.

### Setup for Outcome (Performance Measures)

Within the simulation framework described above, we modeled 3 key outcomes to assess program effectiveness. The generation of these outcomes for each user-program interaction followed the relationships defined in “Setup for the Relationship Between Reward and Context” (differing based on whether it was an AI Call or CHS-based simulation). We compared the average value of each outcome measure over 100 repetitions, each involving 3000 user-item interactions (call selections) with a randomly shuffled sequence of users. The three specific outcome measures we used are:

1. Call success: modeled using a logistic linear model, where a higher success rate (completion of the call) indicated a more desirable outcome. The probability of success for a given user and call type was determined by the model described in “Setup for the Relationship Between Reward and Context.”
2. Patient Health Questionnaire-2 (PHQ-2) Score [19]: modeled using a linear model, where lower scores on the PHQ-2, a measure of depressive symptoms, indicated better mental well-being. The predicted PHQ-2 score depended on user context and program assignment according to the relationships in “Setup for the Relationship Between Reward and Context.” While the underlying relationship was modeled linearly, the final simulated PHQ-2 outcome for an interaction was generated as an integer using probabilistic rounding based on the linear prediction, reflecting the discrete nature of the scale.
3. Self-Reported Health (SRH) [18]: modeled using a linear model, with higher scores on the 5-point SRH scale (1 being “very bad” and 5 being “very good”) representing better perceived health status. Similar to PHQ-2, the predicted SRH score was a function of user and program characteristics as defined in “Setup for the Relationship Between Reward and Context.” The final simulated SRH outcome was generated as an integer on the 5-point scale using probabilistic rounding based on the linear model’s prediction.

### Evaluation Methods

Four allocation methods were evaluated based on 2 metrics. The first metric was the distribution of the average outcome across simulation runs. For each of the 3 performance measures, call success, PHQ-2 score, and SRH, the average value of the outcome for each of the 4 allocation methods was calculated. This represents the average scores of the subjects who received the care call according to each allocation rule.

The second metric was average cumulative regret over time, or with the increasing number of subjects. Cumulative regret represents the loss incurred from selecting a suboptimal arm over time. For the first 2 allocation methods, regret never decreases, and cumulative regret increases linearly over time. For Method 3, both regret and cumulative regret are zero. For the proposed method, RL-based precision digital care, if the method learns the optimal arm, cumulative regret tapers off after increasing initially. The tapering nature indicates that the method is learning the optimal arm.

### Ethical Considerations

This study was reviewed and determined to be exempt from ethical approval by the Institutional Review Board of Seoul National University (E2408/004-007; August 14, 2024), as it used publicly available and deidentified data that did not involve direct interaction with human participants. The study analyzed anonymized AI care call data and aggregated data from the Community Health Survey, and no personally identifiable information was accessed or retained. Because the study used deidentified secondary data and simulation-based analyses, informed consent from participants was not required. All procedures were conducted in accordance with the Declaration of Helsinki.

## Results

**Figure 1:**
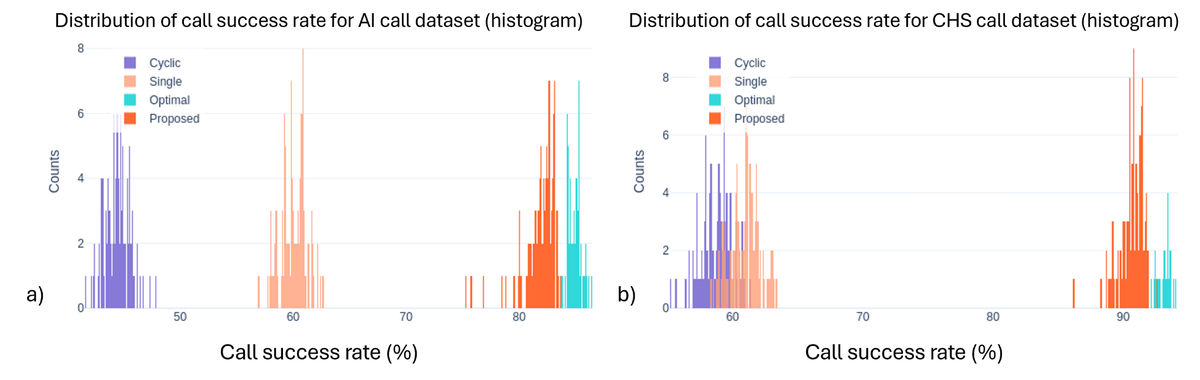
Distribution of average call success rates across the four allocation methods for (a) AI call dataset and (b) community health survey dataset. The proposed precision digital care, a reinforcement learning-based approach, achieves superior performance in call success rates compared to systematic and single best allocation approaches. Note: “Cyclic” in the figure legend refers to the systematic (baseline) allocation method described in the text. AI: artificial intelligence; CHS: community health survey.

**Figure 2:**
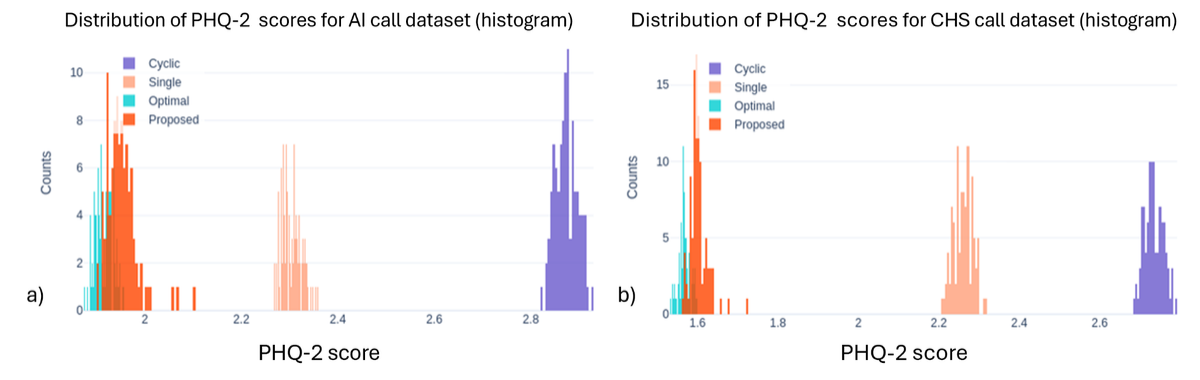
Distribution of average patient health questionnaire-2 scores across the four allocation methods for (a) AI call dataset and (b) community health survey dataset. The proposed precision digital care approach achieves superior performance in patient health questionnaire-2 scores compared to systematic and single best allocation approaches. Note: “Cyclic” in the figure legend refers to the systematic (baseline) allocation method described in the text. AI: artificial intelligence; CHS: community health survey; PHQ-2: Patient Health Questionnaire-2.

**Figure 3:**
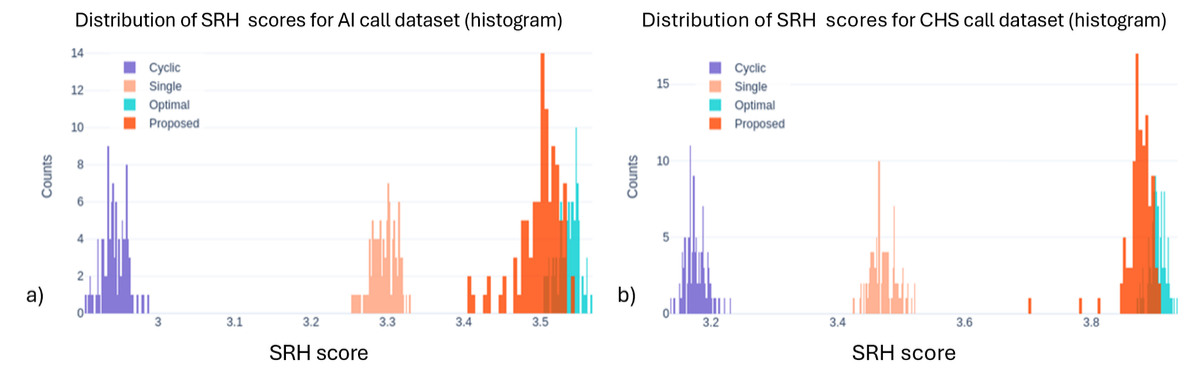
Distribution of average self-reported health scores across the four allocation methods for (a) AI call dataset and (b) community health survey dataset. The proposed approach achieves superior performance in self-reported health scores compared to systematic and single best allocation approaches. Note: “Cyclic” in the figure legend refers to the systematic (baseline) allocation method described in the text. AI: artificial intelligence; CHS: community health survey; SRH: self-reported health.

We compared 4 program allocation methods: systematic, single best, optimal, and our proposed precision digital care with Thompson Sampling, across 3 outcome measures: call success rate, PHQ-2 score (depressive symptoms), and SRH. Figures 1-3 show the outcome distributions for each method over 100 simulation runs, each involving 3000 program assignments. The summary statistics for each outcome, including mean values and standard deviations across 100 simulation runs, are presented in Table 2.

Values represent means (SDs) across 100 simulation runs (n=3000 assignments per run).

Our results demonstrate the significant potential of precision digital care with Thompson Sampling for personalized program allocation. Across all 3 measures, the proposed precision digital care consistently achieved outcomes comparable to the idealized optimal method, which assumes perfect knowledge of individual preferences and program effectiveness. Compared with systematic allocation, the proposed precision digital care approach substantially improved outcomes across all 3 measures. Call success rates increased from 44.45% to 81.86% in the AI Call dataset (84.2% relative improvement) and from 58.72% to 90.68% in CHS (54.4%). PHQ-2 depression scores decreased from 2.87 to 1.95 (32.1% reduction, AI Call) and from 2.73 to 1.60 (41.4%, CHS), while SRH scores improved from 2.94 to 3.50 (19%, AI Call) and from 3.18 to 3.88 (22%, CHS). To compare outcome distributions between systematic allocation and the proposed method, chi-square tests were used for call success rates, and Mann-Whitney *U* tests for PHQ-2 and SRH scores; all comparisons were statistically significant (*P*<.001). These findings indicate that RL-based personalization leads to substantial gains in care effectiveness compared to rule-based or static allocation strategies. These also suggest that precision digital care can effectively learn and adapt to individual needs, leading to near-optimal outcomes even without prior knowledge of individual preferences. As expected, the nonpersonalized baseline methods (“systematic” (cyclic) and “single best”) yielded inferior performances compared to the personalized approaches. This highlights the limitations of assigning programs without considering individual needs and preferences.

**Figure 4:**
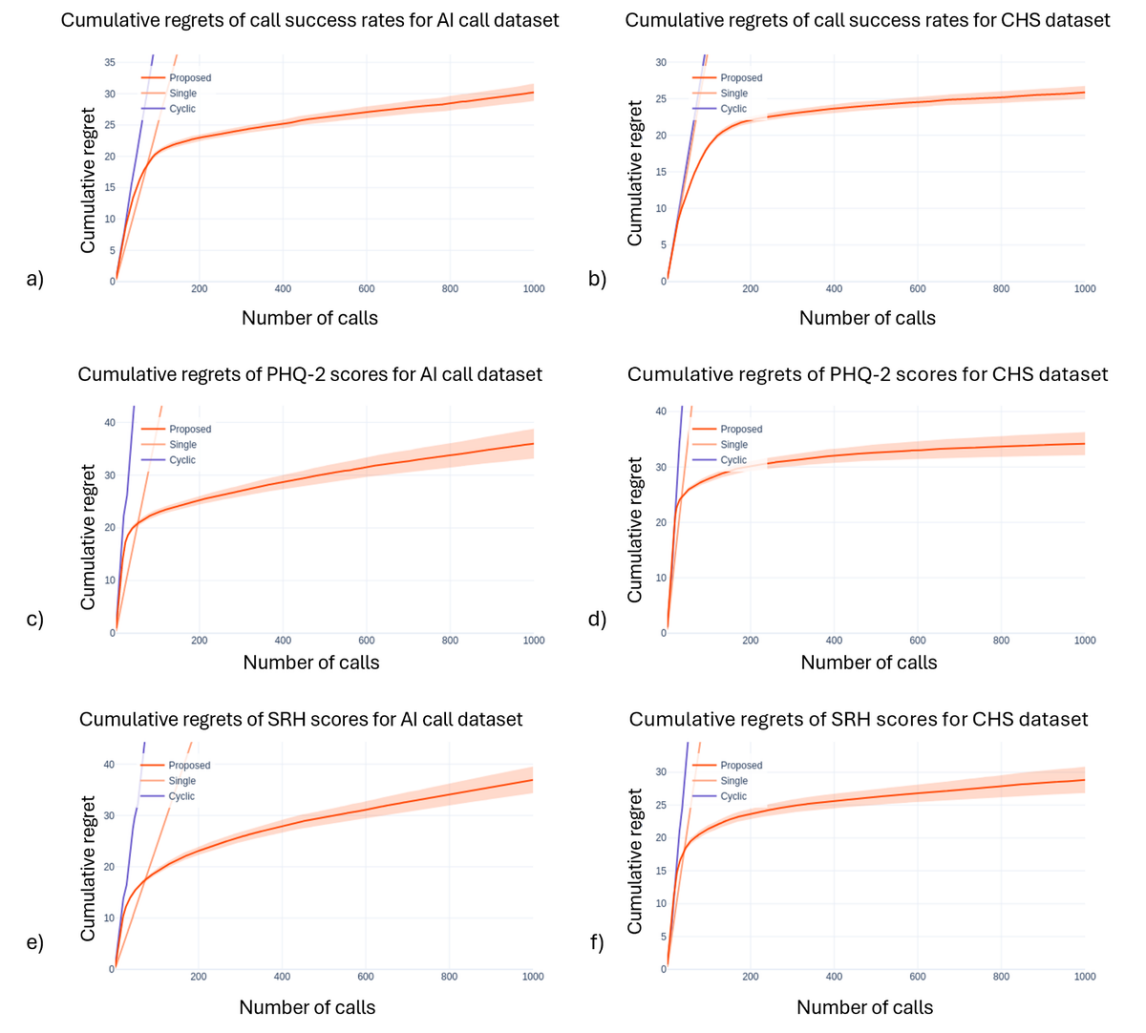
Cumulative regret for (a) call success (AI call dataset), (b) call success (community health survey dataset), (c) Patient Health Questionnaire-2 (AI call dataset) (d) Patient Health Questionnaire-2 (CHS dataset), (e) self-reported health (AI call dataset), and (f) self-reported health (community health survey dataset) models. The rapid decline in regret for precision digital care indicates its ability to efficiently learn and optimize program allocation, significantly outperforming static allocation methods. Note: “Cyclic” in the figure legend refers to the systematic (baseline) allocation method described in the text. AI: artificial intelligence; CHS: community health survey; PHQ-2: Patient Health Questionnaire-2; SRH: self-reported health.

Figure 4 illustrates cumulative regret over time or the number of users. Cumulative regret represents the loss incurred from selecting a suboptimal arm over time. It initially increases rapidly before tapering off, indicating that suboptimal arms are often selected in the beginning, followed by the selection of optimal arms. The figures also show that, depending on the outcome, learning occurs across a varying number of users. Since call success is binary, it takes more users to stabilize the learning process. This pattern suggests that precision digital care rapidly identifies optimal program assignments, reducing the frequency of ineffective interventions as it learns from accumulated user interactions. The stabilization of regret over time indicates that the RL model efficiently converges to near-optimal allocation decisions, ensuring that users receive the most suitable care programs based on their unique characteristics.

These findings underscore the potential of using contextual bandit algorithms like Thompson Sampling for personalized interventions. The ability to dynamically learn and adapt to individual needs can lead to significant improvements, especially when program effectiveness varies greatly across individuals. Overall, these results highlight the effectiveness of precision digital care in dynamically optimizing care program assignments, achieving outcomes close to the idealized optimal method. By leveraging RL, the proposed precision digital care has the potential to continuously improve care services based on user interactions, ultimately enhancing the quality and efficiency of digital health interventions.

**Table 2:** Summary of the simulated care outcomes across allocation methods.

Outcomes and datasets		Systematic allocation (baseline)	Single best	Optimal	Precision digital care (proposed)
**Call success rate^a^**					
	AI^b^ Call, mean (SD)	44.45 (1.12)	59.98 (1.14)	84.89 (0.61)	81.86 (1.45)
	CHS^c^, mean (SD)	58.72 (1.20)	60.94 (1.13)	93.26 (0.48)	90.68 (0.94)
**PHQ-2^d^**					
	AI Call, mean (SD)	2.87 (0.02)	2.3 (0.02)	1.92 (0.02)	1.95 (0.03)
	CHS, mean (SD)	2.73 (0.02)	2.26 (0.02)	1.57 (0.01)	1.6 (0.02)
**SRH^e^**					
	AI Call, mean (SD)	2.94 (0.02)	3.29 (0.02)	3.54 (0.01)	3.5 (0.03)
	CHS, mean (SD)	3.18 (0.02)	3.47 (0.02)	3.9 (0.01)	3.88 (0.02)

^a^Call success rate: mean percentage of successful call completions (higher=better).

^b^AI: artificial intelligence.

^c^CHS: Community Health Survey.

^d^PHQ-2: Patient Health Questionnaire-2; scores range from 0 to 6 (lower=fewer depressive symptoms).

^e^SRH: Self-Reported Health; scores range from 1 (very bad) to 5 (very good) (higher=better health).

## Discussion

### Principal Findings

Our findings highlight the potential of precision digital care with Thompson Sampling in optimizing digital care service allocation for older adults. Through simulation studies, we demonstrated that personalized program allocation leads to improved care outcomes compared to nonpersonalized methods. Specifically, the precision digital care approach outperformed both systematic and single-best allocation approaches, achieving results close to the idealized theoretical upper bound. This underscores the importance of hyper-personalization in digital health care, particularly in aging populations facing growing care demands and workforce shortages.

The outcomes evaluated in this simulation study represent key dimensions of effective care delivery for older adults living alone. Call success indicates sustained engagement with care services and regular monitoring, both critical for timely detection of health or safety concerns and prevention of unattended deaths- an emerging social problem in Korea [20]. PHQ-2 scores represent depressive symptoms that affect daily functioning, social participation, and self-care capacity, while self-reported health reflects subjective well-being and perceived ability to age in place.

In real-world implementation, improvements in these outcomes would support the delivery of responsive, person-centered care. Importantly, precision digital care is designed to augment (not replace) human care relationships. In Korea’s context, direct care workers often face overtime burdens and emotional exhaustion, contributing to workforce shortages [2,3]. The proposed approach optimizes routine care allocation through AI-driven monitoring, allowing human workers to focus on complex cases requiring empathy, clinical judgment, and relational continuity. This human-AI collaboration model addresses workforce sustainability while preserving the irreplaceable value of human connection in care relationships. Rather than automating care relationships, the system automates administrative burden, the repetitive monitoring tasks that overwhelm workers, enabling them to provide higher-quality human care where it matters most. Together, these outcomes support the goal of enabling older adults to age with dignity and autonomy while ensuring appropriate care allocation.

These findings align with previous research emphasizing the importance of personalization in digital health interventions for older adults. A scoping review of digital health interventions targeting older populations found that personalized features improve self-management and psychosocial health, but also noted that experimental studies validating these effects are scarce [21]. Our study contributes to this gap by providing a systematic, AI-driven approach to personalization, demonstrating through simulation that RL can effectively optimize program allocation based on user characteristics. Unlike previous work, which primarily relies on static program designs, our approach adapts dynamically, ensuring that interventions are continually optimized over time.

Our study also relates to research exploring contextual bandit algorithms in mobile health settings, where RL has been used to optimize treatment timing and intervention strategies [22]. Existing studies have demonstrated the feasibility of such approaches in personalized patient engagement and behavior change interventions [23]. However, most prior applications focus on mobile health and preventive care rather than digital older adult care services. Our findings extend these applications by showing that contextual bandits can optimize not just treatment timing but also care program selection for older adults in government-supported digital health initiatives. This represents a novel application of RL in aging-related digital interventions, offering a scalable solution for optimizing care programs at the population level.

Beyond demonstrating the effectiveness of RL for digital care optimization, our findings have broader implications for the scalability of AI-driven decision models in government-funded care programs. As governments invest in digital health solutions to address aging populations and workforce shortages, integrating AI-driven program allocation systems can improve care efficiency without requiring extensive prelabeled datasets [24]. The Korean AI care call program exemplifies this trend, yet current models lack individualized program allocation strategies. By applying the proposed precision digital care approach for personalization of care in such settings, policymakers could transition from standardized digital interventions to truly personalized care, ensuring that digital services maximize impact and cost efficiency. More broadly, the integration of AI-driven decision-making into digital care services aligns with global trends toward precision health and personalized medicine, advocating for a shift away from static care delivery models.

Despite these promising findings, several limitations must be acknowledged. This study relied on synthetic data and simulations, rather than real-world implementation. Although the data was constructed using existing survey datasets and AI care call user characteristics, real-world trials are necessary to validate these findings in practice. Additionally, the current model was developed within the Korean digital care context, and further research is needed to assess the adaptability of precision digital care in different health care systems and cultural settings.

This study’s focus on low-income, socially isolated older adults living alone reflects an intentional equity commitment. These populations face compounded vulnerabilities, limited access to family support, health care services, and social resources, placing them at increased risk of adverse outcomes [25]. Precision digital care has particular promise for reducing geographic and functional disparities. Our simulations incorporated domain-informed interaction effects in which rural users and those with functional limitations were modeled as benefiting from different care configurations. For example, based on observed patterns in Korean older adult care contexts, rural older adults were modeled as preferring more frequent calls (potentially compensating for limited in-person service access), while those with functional difficulties were assumed to benefit from longer interaction times (allowing more comprehensive needs assessment). Similarly, older adults with cognitive limitations were modeled as engaging better with simpler check-in style calls rather than complex health information content.

By identifying and responding to these differential needs, precision digital care can help bridge urban-rural care gaps, accommodate varying cognitive and functional capacities, and ensure that digital care innovations reduce rather than exacerbate existing inequities. However, realizing this potential requires careful attention to digital literacy barriers, technology access, and culturally appropriate implementation, critical considerations for future real-world validation studies.

Beyond technical and operational concerns, AI-driven personalization in digital health raises ethical considerations, such as potential bias in program allocation, equity in digital access, and privacy concerns related to AI-driven recommendations [26]. Future studies should explore fairness-aware RL approaches to ensure equitable care distribution, particularly for older adults who may have limited digital literacy or reduced access to technology. Furthermore, while RL-based precision digital care optimizes individualized program assignments, its integration into broader multilevel care frameworks, such as clinical care coordination, home-based services, and telemedicine ecosystems, remains an open challenge. Ensuring seamless interoperability with existing digital health platforms will be essential for widespread adoption.

To address these limitations, we plan to conduct real-world validation studies in a publicly funded AI care call program in Korea, assessing precision digital care’s impact on user satisfaction, health outcomes, and long-term engagement, with an expanded set of objective measures (eg, health care use and device-measured activity) to complement self-reports. Although equity was not quantitatively evaluated in this study, the intervention setting, a publicly funded AI care call program for low-income, socially vulnerable older adults living alone, reflects an inherent focus on underserved populations. Future research should also explore hybrid AI models, integrating deep learning and RL to further refine personalized digital interventions. Additionally, a critical next step is to examine how RL-based precision digital care performs across diverse populations, ensuring that AI-driven personalization remains both effective and equitable across different health care systems.

### Conclusions

This study introduced precision digital care, an AI-driven RL model for optimizing personalized digital care service allocation. Our simulation-based evaluation demonstrated that precision digital care significantly improves care outcomes by dynamically adapting interventions to individual user characteristics. These findings highlight the potential of AI-driven personalization in enhancing the efficiency and effectiveness of digital health interventions, particularly in aging populations where scalable, individualized care is increasingly needed.

As digital care programs expand, ensuring personalized, data-driven decision-making will be critical in maximizing their impact. Precision digital care offers a scalable approach to deliver dignified, person-centered care at scale. By using RL to understand and respond to individual needs, the proposed framework can help older adults maintain autonomy, social connection, and well-being while supporting sustainable care systems. The ultimate goal is not simply algorithmic optimization but improving lived experiences and quality of life in later years through adaptive, humane digital care that honors individual dignity and preferences. However, further real-world validation is required to confirm the effectiveness of precision digital care beyond simulations and to address practical implementation challenges.

Future research should focus on scaling and adapting precision digital care to diverse health care settings, ensuring ethical AI deployment, and integrating hybrid AI models to enhance personalization. Establishing fair, transparent, and explainable AI systems will be key to promoting trust and equitable access in digital health services for aging populations worldwide.

## Abbreviations

AI: artificial intelligence
CHS: community health survey
PHQ-2: Patient Health Questionnaire-2
RL: reinforcement learning
SRH: self-reported health

## Data Availability

The datasets generated and/or analyzed during this study are not publicly available due to Institutional Review Board regulations, but are available from the corresponding author on reasonable request.
